# On the Antagonism of Atropia and Morphia, Founded upon Observations and Experiments Made at the U.S.A. Hospital for Injuries and Diseases of the Nervous System

**Published:** 1865-11

**Authors:** S. Weir Mitchell, Wm. W. Keen, Geo. R. Morehouse


### ON THE ANTAGONISM OF ATROPIA AND MORPHIA, FOUNDED UPON OBSERVATIONS AND EXPERIMENTS MADE AT THE U.S.A. HOSPITAL FOR INJURIES AND DISEASES OF THE NERVOUS SYSTEM.

By S. WEIR MITCHELL, M.D., WM. W. KEEN, M.D., and GEO. R. MOREHOUSE, M.D.

During our connection with the U.S.A. Hospital for Injuries and Diseases of the Nervous System, we have been obliged to resort to every possible expedient for soothing the pain of those terrible cases of neuralgia, which, in some shape, are apt to follow as a consequence of neural injuries. Among these means, incessant use has been made of hypodermic injections, which alone, in many instances, seemed able to overcome the anguish of certain forms of neuralgic distress. To what extent we have employed this mode of relief may be gathered from the fact that, at certain periods of our service, the resident surgeons made every day from twenty to thirty subcutaneous injections. In one case, half a grain to a grain of morphia was injected thrice a day, and the man finally recovered, after having used nearly four hundred injections.

We were naturally led to examine with care into the pretensions of the several agents which have credit for their power to lessen or destroy the sense of pain. The results of this inquiry were of the more value, because they were confined to the use of these agents by injections only, and because they were studied by more than a single observer. Our investigation brought us finally to consider the therapeutic relations of atropia and morphia, to which subject the greater bulk of this paper will be devoted.

The information which our note-books give in regard to the comparative value of remedies used to allay pain, is the result of an almost unexampled experience, and we shall not hesitate briefly to relate it before passing on to our main topic.

After repeated trials of conia, atropia, and datura, with the intention of relieving pain by their subdermal use, we ceased to resort to them. On the other hand, the employment of morphia, or of some preparation of opium for subcutaneous use, became a part of the every-day routine of practice.

Like others, we have met with certain inconveniences attendant upon this mode of employing morphia. In rare cases it always caused distressing sick stomach, but as the pain for which we used it was oftentimes agonizing, the patient usually preferred to endure the sick stomach rather than fail of the delightful relief he obtained from the injection. In these instances it was commonly observed that the morphia ceased after a time to produce either nausea or emesis.

The local annoyances resulting from injections so long continued and so numerous, were sometimes very embarassing, for though in some men they could be used in the same limb week after week, in others the numerous punctures produced a very unpleasant increase of sensitiveness in the part. Such an instance may be found on page 151, Case 31, of our treatise on wounds and other injuries of the nerves. In other persons, the injections gave rise to occasional abscesses, and in a soldier who was at one and the same time the subject of a very painful wound of the arm, and of a cold abscess on the back, every injection gave rise to a large indolent abscess. One instance of erysipelas following the use of an injection was seen by us. (*Op. cit. p.* 150.)

As the opinion of many good observers is quite decided as to the fact that the injection gives the same relief, whether made near to or remote from the seat of pain, we may with reason be asked, why we used so many injections in the same limb or neighborhood. The answer lies in the fact that our patients very early, and we, ourselves, later and more reluctantly, reached the conclusion that the point at which the injection was to be employed was not a matter of indifference. In the milder instances of neuralgia, a subdermal injection of morphia used anywhere in the body did give relief, but in cases of "burning neuralgia," such as we have described in our book on nerve wounds, *p.* 100, *et seq.*, the nearer we could bring the agent to the place where the pain was felt, the greater was the ease obtained. We are the more anxious to insist upon this matter, because we neglected to make the same comment when detailing our mode of treating these lesions in the volume above mentioned. The belief thus reached, is certainly not altogether unphysiological, as we very well know that morphia is capable of causing a local paralysis of sensory nerves, with which it may come in contact. * * * * *

If we be correct in the views expressed in the foregoing pages, certain practical lessons of some value may be learned from them.

If atropia lessens or destroys the unpleasant influence of morphia on the cerebrum, but does not alter its power to allay pain, there seems no reason why we should not use them together so as to obtain all that is best from the morphia with the least amount of after discomfort.

We have certainly had good results from such a use of both drugs, in the form of suppositories, in cases of disease of the bladder or generative organs.

Again, it is sometimes desirable to use either drug in very full doses. This we may do quite fearlessly, when assured of our ability to restrain its action by a full exhibition of its opponent.

The foregoing experiments and observations authorize us, we think, to draw the following conclusions as to the use of hypodermic injections, and as to the antagonism of atropia and morphia:—

1. Conia, atropia, and daturia have no power to lessen pain when used subdermally.

2. Morphia thus used is of the utmost value to relieve pain, and is most potent, in certain forms of neuralgia, the nearer it is applied to the seat of the suffering.

3. Morphia lowers the pulse slightly, or not at all; atropia usually lowers the pulse a few beats within ten minutes, and then raises it twenty or fifty beats within an hour. The pulse finally falls about the tenth hour below the normal number, and regains its healthy rate within twenty-four hours.

4. Morphia has no power to prevent atropia from thus influencing the pulse, so that, as regards the circulation, they do not counteract one another.

5. During the change of the pulse under atropia, the number of respirations is hardly altered at all.

6. As regards the eye, the two agents in question are mutually antagonistic, but atropia continues to act for a much longer time than morphia.

7. The cerebral symptoms caused by either drug are, to a great extent, capable of being overcome by the other, but owing to the different rates at which they move to affect the system, it is not easy to obtain a perfect balance of effects, and this is made the more difficult from the fact, already mentioned that atropia has the greater duration of toxic activity.

8. The dry mouth of atropia is not made less by the coincident or precedent use of morphia. Atropia does not constipate, and may even relax the bowels; morphia has a reverse tendency.

9. The nausea of morphia is not antagonized or prevented by atropia.

10. Both agents cause dysuria in certain cases, nor is the dysuria occasioned by the one agent relieved by the other.

11. Atropia has no ability to alter or lessen the energy with which morphia acts to diminish sensibility or relieve the pain of neuralgic disease.

12. As regards toxic effects upon the cerebral organs, the two agents are mutually antidotal, but this antagonism does not prevail throughout the whole range of their influence, so that, in some respects, they do not counteract one another, while, as concerns one organ, the bladder, both seem to affect it in a similar way.—*Amer. Jour. of the Med. Sciences, and Boston Med. and Surg. Jour.*

